# STN7 is not essential for developmental acclimation of *Arabidopsis* to light intensity

**DOI:** 10.1111/tpj.16204

**Published:** 2023-04-11

**Authors:** Sarah E. Flannery, Federica Pastorelli, Thomas Z. Emrich‐Mills, Stuart A. Casson, C. Neil Hunter, Mark J. Dickman, Philip J. Jackson, Matthew P. Johnson

**Affiliations:** ^1^ Plants, Photosynthesis and Soil, School of Biosciences University of Sheffield Firth Court, Western Bank Sheffield UK; ^2^ Department of Chemical and Biological Engineering University of Sheffield Sheffield UK

**Keywords:** acclimation, proteomics, thylakoid, light harvesting, electron transfer, photosynthesis, photosystem, *Arabidopsis thaliana*, signalling

## Abstract

Plants respond to changing light intensity in the short term through regulation of light harvesting, electron transfer, and metabolism to mitigate redox stress. A sustained shift in light intensity leads to a long‐term acclimation response (LTR). This involves adjustment in the stoichiometry of photosynthetic complexes through *de novo* synthesis and degradation of specific proteins associated with the thylakoid membrane. The light‐harvesting complex II (LHCII) serine/threonine kinase STN7 plays a key role in short‐term light harvesting regulation and was also suggested to be crucial to the LTR. Arabidopsis plants lacking STN7 (*stn7*) shifted to low light experience higher photosystem II (PSII) redox pressure than the wild type or those lacking the cognate phosphatase TAP38 (*tap38*), while the reverse is true at high light, where *tap38* suffers more. In principle, the LTR should allow optimisation of the stoichiometry of photosynthetic complexes to mitigate these effects. We used quantitative label‐free proteomics to assess how the relative abundance of photosynthetic proteins varied with growth light intensity in wild‐type, *stn7*, and *tap38* plants. All plants were able to adjust photosystem I, LHCII, cytochrome *b*_6_ *f*, and ATP synthase abundance with changing white light intensity, demonstrating neither STN7 nor TAP38 is crucial to the LTR per se. However, *stn7* plants grown for several weeks at low light (LL) or moderate light (ML) still showed high PSII redox pressure and correspondingly lower PSII efficiency, CO_2_ assimilation, and leaf area compared to wild‐type and *tap38* plants, hence the LTR is unable to fully ameliorate these symptoms. In contrast, under high light growth conditions the mutants and wild type behaved similarly. These data are consistent with the paramount role of STN7‐dependent LHCII phosphorylation in tuning PSII redox state for optimal growth in LL and ML conditions.

## Significance Statement

The light harvesting complex II (LHCII) serine/threonine kinase STN7 has been previously implicated in both short‐term light harvesting regulation and the long‐term acclimation response (LTR). Here, using label‐free quantitative proteomics we show that plants lacking STN7 are capable of adjusting the stoichiometry of photosynthetic complexes via the LTR, but still suffer a growth penalty in low and moderate light resulting from the loss of light harvesting regulation.

## INTRODUCTION

Plants show a remarkable ability to adapt to different environmental conditions including wide variations in light intensity, spectral quality, temperature, and water availability (reviewed in Walters, 2005; Schöttler & Tóth, 2014). A key element of this flexibility is the short‐term (regulatory) and long‐term (acclimation) adaptation strategies that adjust the photosynthetic light reactions occurring within the chloroplast thylakoid membrane to match the requirements of the downstream metabolism. The short‐term regulatory processes seek to mitigate the consequences of a sudden shift in environmental conditions that upset homeostasis, thereby avoiding photo‐oxidative stress (Li et al., 2009). Short‐term regulatory mechanisms include (i) non‐photochemical quenching (NPQ), the photoprotective dissipation of excess excitation energy as heat (Murchie & Ruban, 2019); (ii) photosynthetic control (Malone et al., 2021), the moderation of electron transfer between cytochrome *b*_6_ *f* (cyt*b*_6_ *f*) and photosystem I (PSI); (iii) state transitions (Goldschmidt‐Clermont & Bassi, 2015), balancing the relative rate of excitation of PSI versus photosystem II (PSII); (iv) the PSII repair cycle (Theis and Schroda, 2016), the proteolytic degradation of photo‐damaged PSII and its replacement with fresh protein; and (v) dynamic thylakoid stacking, the reversible changes in the number of grana per chloroplast, the number of membrane layers per grana, and grana diameter (Johnson & Wientjes, 2020). In contrast, the long‐term acclimation response (LTR) seeks to adjust the stoichiometry of the photosynthetic complexes through *de novo* synthesis or degradation to establish homeostasis under the prevailing conditions (Anderson, 1986). The LTR process can be further divided in Arabidopsis into (i) dynamic acclimation, where *de novo* synthesis and degradation of specific proteins modifies the composition of the chloroplast (Athanasiou et al., 2010; Suorsa et al., 2012; Walters & Horton, 1994; Yin & Johnson, 2000), and (ii) developmental acclimation, wherein leaf anatomy is altered in addition to changes in the chloroplast proteome (Anderson, 1986; Anderson et al., 1988; Bailey et al., 2001; Bailey et al., 2004; Boardman, 1977; Schöttler & Tóth, 2014; Vialet‐Chabrand et al., 2017; Walters, 2005). The LTR to low light (LL) in Arabidopsis involves the increased synthesis of PSI and light‐harvesting complex II (LHCII) proteins relative to PSII together with increased thylakoid membrane grana stacking, while the LTR to high light (HL) involves decreased PSI and LHCII levels relative to PSII and increased levels of ribulose‐1,5‐bisphosphate carboxylase/oxygenase (RuBisCO), ATP synthase, and cyt*b*_6_ *f* and decreased membrane stacking (Bailey et al., 2001; Bailey et al., 2004; Ballottari et al., 2007; Flannery et al., 2021a; Schumann et al., 2017; Vialet‐Chabrand et al., 2017; Walters, 2005; Walters & Horton, 1994; Ware et al., 2015; Wientjes et al., 2013a; Wientjes et al., 2013b).

The signalling pathways responsible for provoking the LTR remain under investigation; however, key roles have been described for the redox state of the thylakoid plastoquinone electron acceptor pool (PQ pool) (2005b; Brautigam et al., 2009; Fey et al., 2005a; Frigerio et al., 2007; Huner et al., 1996; Pfannschmidt et al., 1999; Rosso et al., 2009; Zoulias et al., 2021), PSI redox state via the thioredoxin/ferredoxin system (Trx/Fd) (2005b; Adamiec et al., 2008; Fey et al., 2005a; Piippo et al., 2006), the glucose‐6‐phosphate/phosphate translocator GPT2 (Athanasiou et al., 2010), and more recently the photoreceptor protein phytochrome B (phyB) (Ibrahim et al., 2022). One area of cross‐over between short‐ and long‐term responses is the LHCII serine/threonine kinase STN7 and its cognate phosphatase TAP38 (Bellafiore et al., 2005; Pesaresi et al., 2009, 2011; Pribil et al., 2010; Shapiguzov et al., 2010). STN7 is itself regulated by a complex network of interactions involving inputs from both the light reactions and chloroplast stromal metabolism. STN7 is activated by binding of PQH_2_ to the oxidising site (Q_p_) of the cyt*b*_6_ *f* complex, inactivated by PQ pool oxidation, and inhibited by build‐up of ΔpH and/or reduced Trx (Fernyhough et al., 1984; Rintamäki et al., 2000; Vener et al., 1997). In Arabidopsis, STN7 activity is also correlated with the phosphorylation state of the S526, T537, and T541 residues in its own stromal C‐terminal domain, possibly via autocatalysis (Trotta et al., 2016; Willig et al., 2011). Phosphorylation of T537 and T541 has been further shown to confer resistance to proteolytic degradation (Trotta et al., 2016). In addition to autophosphorylation, STN7 phosphorylates a range of client proteins, including LHCB1 and LHCB2, components of the major trimeric LHCII complex, LHCB4.2, TSP9 (an unstructured 9‐kDa thylakoid protein of unknown function), FNR1, ribosomal protein S7, and CLPP3 (Bellafiore et al., 2005; Pesaresi et al., 2011; Schönberg et al., 2017; Tikkanen et al., 2006). Amongst these STN7 clients, phosphorylation of N‐terminal domain Thr side chains in the LHCII proteins is best understood. Phospho‐LHCII (pLHCII) is known to modify the protein–protein interactions both laterally within the thylakoid membrane and between membrane layers within the stacked grana regions (Grieco et al., 2015; Kouril et al., 2005; Kyle et al., 1983; Pietrzykowska et al., 2014; Tikkanen et al., 2008). Specific interactions between pLHCII and PSI occur leading to their association at the periphery of the grana in the unstacked stromal lamellae regions, transitioning the system from State I to State II (Pan et al., 2018). These dynamic changes to thylakoid structure modify grana stacking (dynamic thylakoid stacking) to mediate state transitions and influence photosynthetic control and so the PSI acceptor side redox state through partition of electron carriers between cyt*b*_6_ *f* and PSI (Hepworth et al., 2021; Wood et al., 2018, 2019. Unlike the wild type (WT), an Arabidopsis mutant lacking STN7 (*stn7*) shows constitutively larger grana and is unable to perform dynamic thylakoid stacking or state transitions (Bellafiore et al., 2005; Hepworth et al., 2021; Wood et al., 2018, 2019). In addition to affecting these short‐term regulatory processes, STN7 has been suggested to play a key role within the signalling network controlling the LTR by phosphorylating an unidentified downstream protein, (Pesaresi et al., 2009, 2011) which ultimately regulates the expression of photosynthesis‐related genes. Accordingly, the Arabidopsis *stn7* mutant was found to be unable to adjust the relative stoichiometry of LHCII/PSII and PSI/PSII in response to growth under light of varying spectral quality (Bonardi et al., 2005; Pesaresi et al., 2009). Indeed, *stn7* shows a growth defect compared to WT, particularly under fluctuating light, and plants grown under moderate light (ML) show lower CO_2_ assimilation when switched to LL (Bellafiore et al., 2005; Grieco et al., 2012; Hepworth et al., 2021).

In contrast to STN7, less is known about the regulation of TAP38 (also known as PPH1), which dephosphorylates LHCII, but whose specificity towards other STN7 clients remains to be established. Unlike STN7, TAP38 appears to be constitutively active and not subject to redox control (Pribil et al., 2010; Shapiguzov et al., 2010). An Arabidopsis mutant lacking TAP38 (*tap38*) shows constitutively smaller grana and is also unable to perform dynamic thylakoid stacking or state transitions (Armbruster et al., 2013; Wood et al., 2019). Unlike the *stn7* mutant, *tap38* also showed 30–40% more growth compared to WT under LL (Pribil et al., 2010); however, when exposed to HL *tap38* plants showed lower CO_2_ assimilation compared to both WT and *stn7* in addition to more reduced PSI and PSII acceptor sides (Hepworth et al., 2021). The extent to which the growth and CO_2_ assimilation phenotypes in *stn7* and *tap38* are primarily related to a failure of short‐ or long‐term adaptation strategies remains unclear. In this study we combine label‐free quantitative proteomics (Flannery et al., 2021a, 2021b) with photosynthetic phenotyping using structured illumination microscopy, electron microscopy, chlorophyll fluorescence, and absorption spectroscopy to test the ability of the *stn7* and *tap38* mutants to acclimate to long‐term growth under LL, ML, or HL conditions.

## RESULTS

### *stn7* plants show reduced growth and CO_2_ assimilation in LL and ML while *tap38* plants show enhanced growth at LL compared to WT

**Figure 1:**
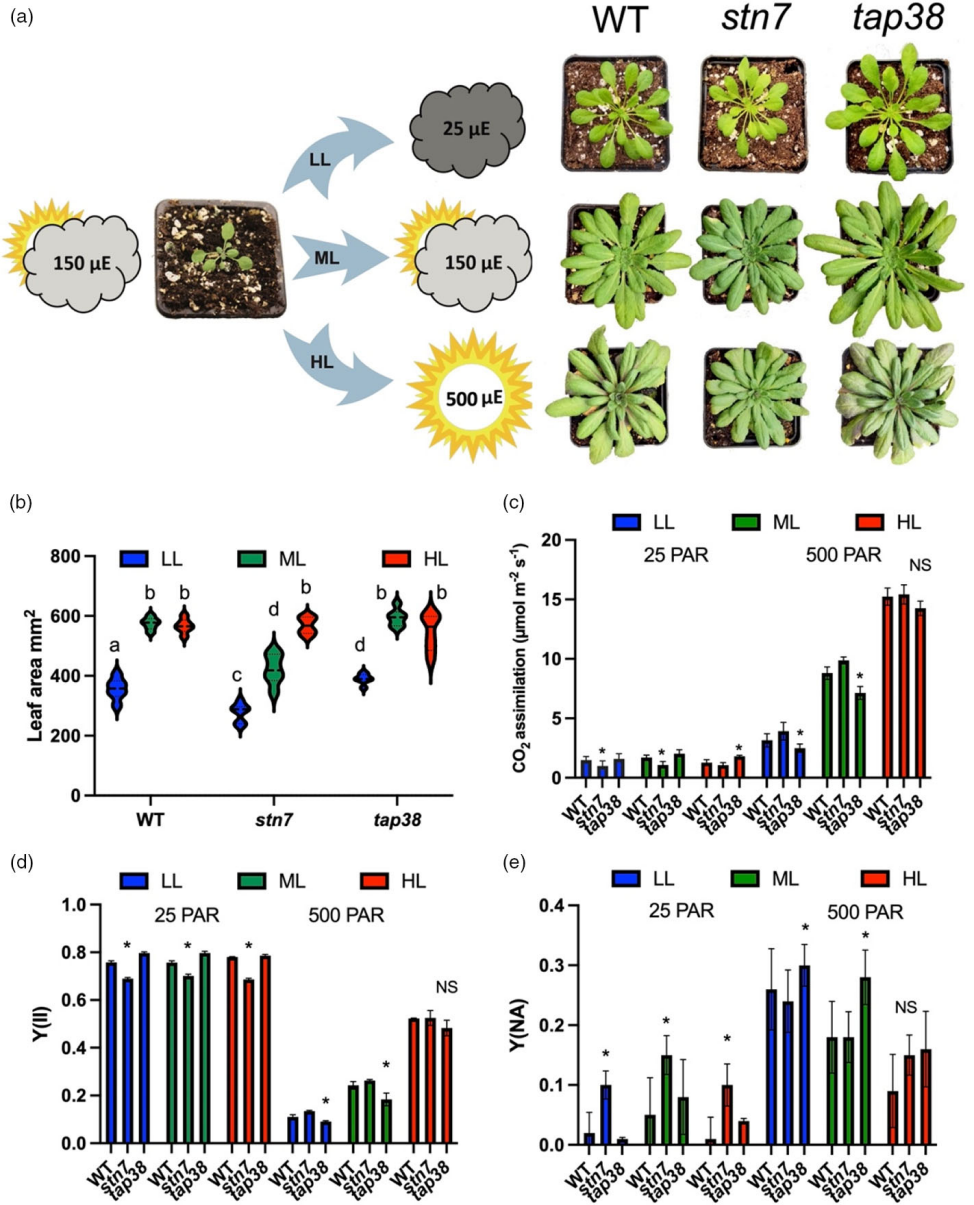
*stn7* plants show reduced growth and CO_2_ assimilation in LL and ML and *tap38* plants show enhanced growth at LL compared to WT. (a) Plants were initially grown under moderate light (ML, Step 1) at 150 μmol photons m^−2^ s^−1^ and then acclimated (Step 2) under low light (LL) at 25 μmol photons m^−2^ s^−1^ or high light (HL) at 500 μmol photons m^−2^ s^−1^ (HL) or maintained under ML conditions (see Methods). The plants were photographed at 2 weeks post‐Step 1 and at 7 (LL), 5 (ML), and 4 (HL) weeks post‐Step 2. (b) Leaf area (mm^2^) of plants following 7 (LL), 5 (ML), or 4 (HL) weeks of growth shown as violin plots (*n* = 15). Pairwise significant differences between light intensity/genotype experiments, calculated using one‐way anova with Tukey's multiple comparisons post hoc test are: a/b (*P* ≤ 0.0001), a/c (*P* = 0.0011), b/c (*P* ≤ 0.0001), a/d (*P* = 0.0013), b/d (*P* = 0.0021), and c/d (*P* = 0.0022). (c) CO_2_ assimilation. (d) PSII quantum yield (Y(II)). (e) PSI acceptor side limitation (Y(NA)). All values in panels (c–e) were measured at either 25 or 500 μmol photons m^−2^ s^−1^ (PAR) using 635 nm LEDs. In panels (c)–(e), * denotes significant differences with respect to WT at each light intensity as determined by a modified Welch's *t*‐test (*n* = 4, *q* < 0.05).

Col‐0 (WT), *stn7*, and *tap38* Arabidopsis plants were grown with an 8‐h photoperiod for 2 weeks under moderate white light (ML; 150 μmol photons m^−2^ s^−1^ [PAR]; emission spectrum shown in Figure S1) and then either moved to LL (25 PAR) or HL (500 PAR) or kept at ML as previously described (Flannery et al., 2021a). Unlike previous studies, which employed coloured lights which preferentially excite PSI or PSII (e.g. Pesaresi et al., 2009), here we purposely used broadband white light conditions to avoid pushing the system to extremes. Representative images of the plants at 5 weeks post‐germination are shown in Figure 1a. As previously reported, *stn7* plants show a decreased chlorophyll *a*/*b* ratio under LL and ML, whereas *tap38* plants show an increased ratio compared to the WT (Table 1) (Bellafiore et al., 2005; Pribil et al., 2010; Tikkanen et al., 2006). Under LL conditions, *tap38* plants were significantly larger 35 days post‐germination relative to WT, which were in turn larger than *stn7* plants, consistent with previous results (Bellafiore et al., 2005: Pribil et al., 2010) (Figure 1a,b), while total chlorophyll content was also enhanced in *tap38* (Table 1). In contrast, *tap38* and WT plants maintained under ML conditions were not significantly different, while *stn7* plants were smaller (Figure 1a,b). However, when grown under HL conditions no significant difference was found between the three types of plant (Figure 1a,b). To understand whether the LL and ML growth phenotypes reflected disruptions to photosynthesis we examined the maximum rate of CO_2_ assimilation for the LL, ML, and HL plants at 25 and 500 PAR, probing acclimation of the LL and HL plants and assessing the responses of ML plants upon a shift to these conditions (Figure 1c). At 25 PAR, in LL and ML grown plants CO_2_ assimilation capacity was higher in WT and *tap38* compared to *stn7.* Whereas for HL grown plants it was higher in *tap38* compared to WT and *stn7* at 25 PAR (Figure 1c). At 500 PAR, CO_2_ assimilation capacity was ordered as follows in all plants: HL > ML > LL, consistent with the acclimation of the plants to light intensity. However, CO_2_ assimilation capacity was higher at 500 PAR for LL and ML grown *stn7* and WT plants compared to *tap38*, though for HL grown plants there was no significant difference (Figure 1c). A similar picture was found for the quantum yield of PSII (Y(II)) determined by chlorophyll fluorescence, with higher yields at 25 PAR for WT and *tap38* relative to *stn7* whether grown at LL, ML, or HL and higher yields for WT and *stn7* at 500 PAR for LL and ML grown plants, although not those grown at HL compared to *tap38* (Figure 1d). The lower Y(II) at 25 PAR in *stn7* plants and at 500 PAR in *tap38* plants could be traced to a more reduced PSII acceptor side (as inferred by the parameter 1 − qP) in each case (Figure S2a). NPQ in contrast was higher in *stn7* and WT compared to *tap38* at 500 PAR in LL, ML, and HL grown plants (Figure S2b). PSI quantum yield (Y(I)) was also higher at 25 PAR in *tap38* and WT LL, ML, and HL grown plants compared to *stn7*, while at 500 PAR *stn7* plants had a relatively higher Y(I) for LL and ML grown plants (Figure S2c). As with PSII, lower PSI yield for *tap38* at 500 PAR and for *stn7* at 25 PAR was traced to higher PSI acceptor side limitation (Y(NA); Figure 1e). The PSI donor side limitation (Y(ND)) was absent at 25 PAR in all plants and not significantly different at 500 PAR in all plants, except HL grown *stn7*, in which it was lower than in WT and *tap38* (Figure S2d). These results demonstrate that *stn7* cannot completely compensate for the loss of short‐term STN7‐dependent phosphorylation via the LTR when developing under LL and ML. However, the higher 1 − qP and Y(NA) values and lower NPQ values in LL and ML *tap38* plants at 500 PAR are overcome in HL *tap38* plants since they suffer no apparent growth penalty at HL.

**Table 1:** Chlorophyll *a*/*b* ratios and total leaf chlorophyll content of plants grown for 2 weeks at 150 μmol photons m^−2^ s^−1^ and then acclimated under low light (LL) at 25 μmol photons m^−2^ s^−1^, moderate light (ML) at 150 μmol photons m^−2^ s^−1^, or high light (HL) at 500 μmol photons m^−2^ s^−1^ (HL). Pairwise significant differences were calculated using one‐way anova with Tukey's multiple comparisons test.

	Chlorophyll *a*/*b* ratios			Total chlorophyll (mg m^−2^)		
	LL	ML	HL	LL	ML	HL
WT	3.0 ± 0.03^a^	3.0 ± 0.03^a^	3.16 ± 0.03^b^	191 ± 6^a^	213 ± 5^c^	233 ± 9^d^
*stn7*	2.62 ± 0.06^c^	2.62 ± 0.06^c^	3.2 ± 0.03^b^	180 ± 4^a^	204 ± 7^bc^	235 ± 4^d^
*tap38*	3.28 ± 0.03^c^	3.28 ± 0.03^c^	3.64 ± 0.05^d^	202 ± 6 ^b^	214 ± 9^c^	229 ± 10^d^

### *stn7* plants can adjust the stoichiometries of the major photosynthetic complexes in response to light intensity

**Figure 2:**
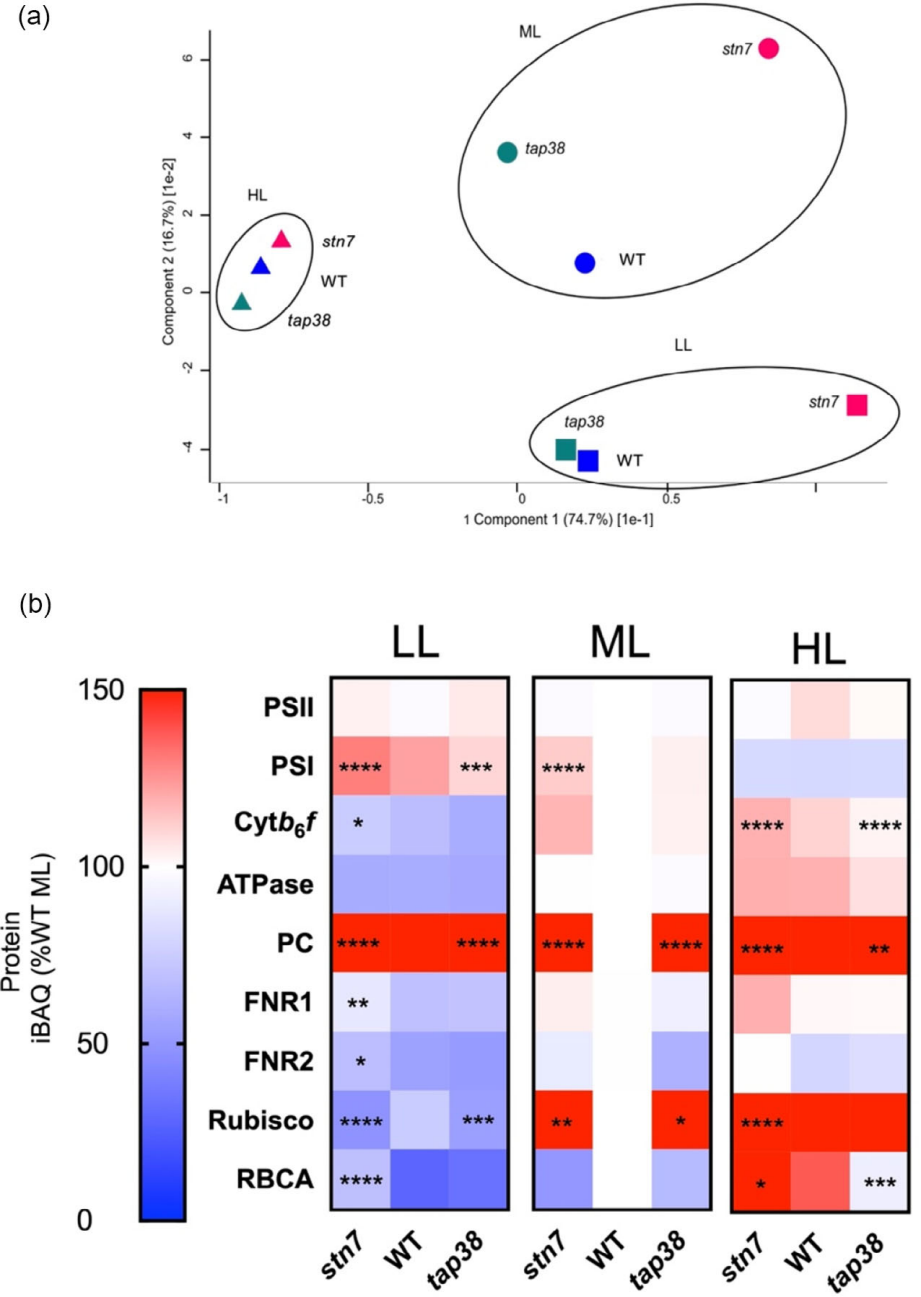
Relative label‐free quantification of the major photosynthetic complexes by mass spectrometry. (a) Principal component analysis of thylakoid protein iBAQ intensities in WT (blue), *stn7* (red), and *tap38* (green) plants grown under LL (squares), ML (circles), and HL (triangles). (b) Heatmap showing the abundances of key photosynthesis‐related proteins and complexes expressed as a percentage of the WT under ML and shaded according to the scale on the left. Each pixel represents the mean of three independent biological replicates, each derived from a pooled thylakoid membrane preparation extracted from 15 plants. The 27 samples were analysed by nanoLC‐MS as three technical repeats (81 analyses in total) in randomised order. Each biological replicate is represented by the median of its three technical repeats. Comparisons of the protein complexes (PSII, PSI, cyt*b*_6_ *f*, and ATP synthase) were made using the sum of the normalised abundance scores of their subunits. Significant differences in relative abundance compared to WT in each light condition, calculated using two‐way anova with Tukey's multiple comparisons post hoc test, are indicated as *P* < 0.05 (*), *P* < 0.01 (**), *P* < 0.001 (***), and *P* < 0.0001 (****).

To investigate whether these differences in growth and photosynthetic traits were caused by a failure of the *stn7* plants to adjust the stoichiometry of the photosynthetic complexes to light intensity, we employed label‐free quantitative (LFQ) mass spectrometry (MS) using a method previously described and validated with orthogonal approaches (Flannery et al., 2021a,b). Three biological replicate thylakoid membrane preparations from each of the WT, *stn7*, and *tap38* plants grown under LL, ML, and HL (27 samples in total) were analysed by nanoLC‐MS as three technical repeats in randomised order. Processing to generate normalised abundance scores for the identified proteins revealed differential protein expression levels. The total number of proteins identified across the 81 analyses was 3216, including 447 quantified proteins mapping to thylakoid membranes (Table S1). Principal component analysis (PCA) of thylakoid protein iBAQ intensities revealed proteomic groupings that were clearly defined according to acclimation to light intensity (Figure 2a). Plants of all three genotypes grown under HL formed the closest grouping, suggesting that both mutants acclimated to HL similarly to the WT. Under LL, the proteomes of WT and *tap38* thylakoids formed a subgroup which, in this case, was markedly distant from the *stn7* mutant. While the ML thylakoids did not associate into any subgroups, WT, *stn7*, and *tap38* all aligned with their LL counterparts along the PC1 axis, indicating that each genotype had shared proteomic features for LL and ML growth that were not present under HL and vice versa. Figure 2b shows heatmaps highlighting the changes in abundance of the major thylakoid photosynthetic complexes and RuBisCO relative to WT ML, with red indicating increases and blue indicating decreases. Proteins significantly different (as determined by two‐way analysis of variance [anova]) in abundance from the WT in each light condition are indicated with asterisks. LFQ analysis revealed that PSII abundance was broadly consistent for all genotypes and growth light intensities in agreement with previous findings (Flannery et al., 2021a). The amount of PSI, however, was more variable, decreasing with increasing growth light intensity in the WT. Contrary to the pattern observed under different spectral profiles (Pesaresi et al., 2009), under varying white light intensity *stn7* showed increased PSI abundance under LL. PSI abundance in ML *stn7* was also significantly higher than in WT at ML, but not significantly different at HL. In *tap38* PSI abundance was significantly lower under LL, although it was unchanged at ML and HL compared to the WT. As expected, cyt*b*_6_ *f* abundance increased in HL and decreased in LL in the WT. In *stn7*, cyt*b*_6_ *f* abundance was increased in LL and HL compared to the WT, but not significantly different under ML. In contrast, *tap38* was only significantly different to the WT under HL, where its abundance was lower. ATP synthase abundance increased with light intensity in the WT, while no significant differences were seen in the mutants compared to the WT. Plastocyanin (PC) content increased in both LL and HL in the WT relative to ML, as reported previously (Flannery et al., 2021a). In both mutants PC abundance was significantly increased in all light conditions compared to the WT. FNR1 content decreased in the WT in LL, although it was similar in ML and HL; the only significant change in the mutants was in LL where FNR1 abundance was higher in *stn7*. Similarly, FNR2 abundance was also increased in LL in *stn7*. Despite being a component of the Calvin–Benson–Bassham cycle, which occurs in the stroma, RuBisCO (RBCL, RBCS‐1A, RBCS‐3B) was identified in our thylakoid membrane preparations, as expected from previous evidence showing it can associate with the thylakoid (Dekker & Boekema, 2005). Some caution should be applied to our quantification of such stromal side associated peripheral proteins, since it cannot be excluded that the affinity of the thylakoid for these proteins also varies with growth light intensity/in the mutants e.g. due to phosphorylation. Nonetheless, RuBisCO shows increased abundance with growth irradiance in the WT, as reported for many other species (Anderson et al., 1988; Schöttler & Tóth, 2014) and consistent with the increased CO_2_ assimilation capacity in HL grown plants (Figure 1c). RBCL is a known STN7 client (Schönberg et al., 2017), and RuBisCO abundance was significantly increased in *stn7* at ML and HL; however, it was also increased in ML in *tap38*. On the other hand, RuBisCO abundance was lower in LL grown *stn7* and *tap38* compared to the WT. RuBisCO activase (RBCA) is involved in the regulation of RuBisCO catalysing the ATP‐dependent dissociation of sugar phosphates from the active site (Spreitzer & Salvucci, 2002). RBCA increases in abundance in the WT with growth irradiance; in the mutants it was higher in *stn7* in LL and HL and lower in HL in *tap38*.

### LHCII protein abundance is increased in *stn7*

**Figure 3:**
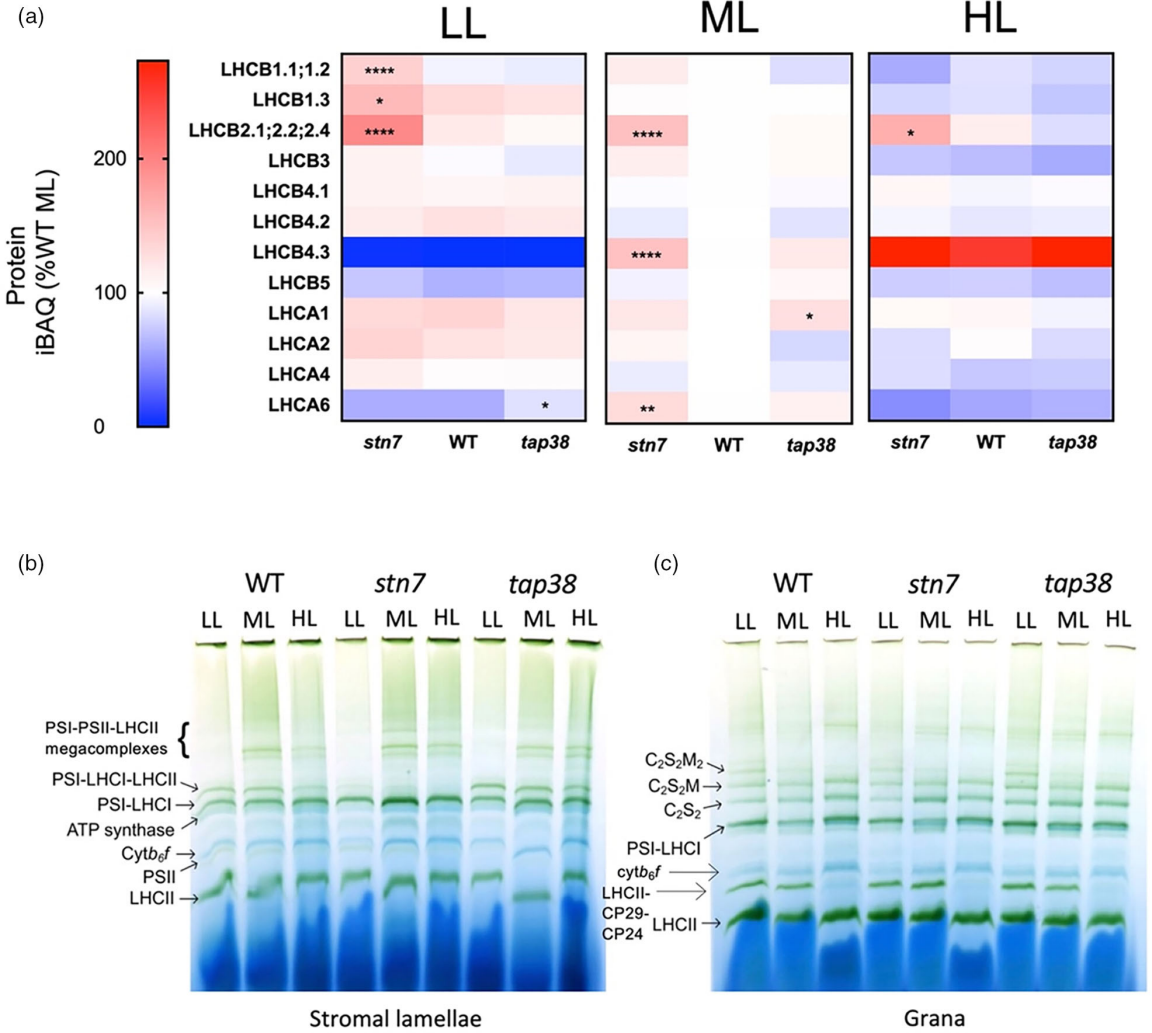
Analysis of PSI and PSII light‐harvesting antenna protein composition by mass spectrometry. (a) Heatmap of the abundances of photosynthetic antenna proteins, expressed as a percentage of the WT at ML. The pixels are shaded and significant differences compared to WT are indicated by asterisks as described in Figure 2. (b, c) BN‐PAGE of solubilised stromal lamellae (b) and granal thylakoid fractions (c).

The effects of growth light intensity and the *stn7* and *tap38* mutations on the relative abundances of the major trimer LHCII components (LHCB1–3), forming the peripheral antenna, and the minor monomeric antenna complexes (LHCB4–6), which constitute the inner antenna of PSII, are shown in Figure 3a. Owing to their sequence identities, LHCB1.1 and LHCB1.2 and similarly LHCB2.1, LHCB2.2, and LHCB2.4 do not produce unique proteotypic tryptic peptides to enable their differentiation. Therefore, the results in Figure 3a represent the combined abundance of the LHCB1.1/1.2 and LHCB2.1/2.2/2.4 isoforms. In the WT, LHCB1.3 and LHCB2.1/2.2/2.4 increased in LL and LHCB1.1/1.3 and LHCB3 decreased in HL similar to previous results (Flannery et al., 2021a). In *stn7*, LHCB1.1/1.2, LHCB1.3, and LHCB2.2/2.3/2.4 levels were significantly increased in LL compared to the WT, although only LHCB2.2/2.3/2.4 expression was significantly higher at ML and HL. In contrast, no significant differences were seen between the WT and *tap38* for these proteins. Of the minor monomeric antenna complexes in the WT, LHCB4.1 is largely unchanged between different growth irradiances, whereas LHCB4.2 increases in LL and decreases in HL, with a similar pattern seen in both mutants. On the other hand, LHCB4.3 decreases strongly in LL and increases strongly in HL in all three genotypes, with relative abundance in *stn7* also significantly elevated under ML. Decreased relative abundance of LHCB5 is seen in both LL and HL in all genotypes, while LHCB6 was not detected.

The basic PSI antenna system in Arabidopsis is comprised of one copy each of LHCA1–4, although it has been shown that PSI can bind additional copies of LHCA1 and LHCA4 under LL (Crepin et al., 2020). Meanwhile, LHCA5 and LHCA6, of lower abundance, are involved in forming the PSI–NDH supercomplex (Peng et al., 2009). LHCA3 and LHCA5 were not detected in this study. Increased abundance of LHCA1 and 2 is seen in the WT in LL, while LHCA4 is stable and LHCA6 expression is decreased in LL. In contrast, under HL the abundance of LHCA4 and LHCA6 decreased and LHCA1 and LHCA2 were not significantly affected (Figure 3a). In LL a similar acclimation pattern was seen in *stn7*, while in ML there was significantly more LHCA6. In *tap38*, the only significant difference with respect to the WT was the slightly higher levels of LHCA6 in LL.

The organisation of photosynthetic complexes was also visualised by BN‐PAGE (Figure 3b,c). First the unstacked stromal lamellae domain of the thylakoid was preferentially solubilised using 1% digitonin (Figure 3b). In the WT the PSI–LHCI–LHCII supercomplex was present at LL and ML but declined in abundance in HL. In contrast, in *stn7* the PSI–LHCI–LHCII supercomplex was virtually absent under all light conditions, though a faint band could be observed under LL, whereas in *tap38* it was present under all light conditions (Figure 3b). Amounts of the ATP synthase and cyt*b*_6_ *f* complexes increased with growth light intensity, while the PSI–PSII–LHCII megacomplexes were absent at LL but present at ML and to a lesser extent at HL. The stacked grana membranes that remained unsolubilised following digitonin treatment were subsequently solubilised using 1% *n*‐dodecyl‐α‐d‐maltoside (Figure 3c). The solubilisation pattern was largely similar in the WT and mutants with a decline in the amounts of the larger C_2_S_2_M_2_ PSII–LHCII supercomplex from LL to HL and an increase in abundance of cyt*b*_6_ *f* with light intensity, while the LHCII–CP24–CP29 subcomplex was absent under HL. The amounts of the free LHCII trimers were similar under each growth light condition (Figure 3c). The differences in the amounts of LHCII phosphorylation between the mutants revealed by total phosphoprotein staining were consistent with the previously reported phenotypes (Figure S3) (Bellafiore et al., 2005; Pribil et al., 2010). *Stn7* plants largely lacked LHCII phosphorylation, while in *tap38* plants phosphorylation was enhanced compared to the WT, particularly in HL samples, while D1/D2 phosphorylation was increased in LL and HL grown plants compared to those grown in ML. TSP9 and PsbH phosphorylation were both enhanced in *stn7*.

### Increased qE‐ and CET‐related protein abundances are found in *stn7* plants

**Figure 4:**
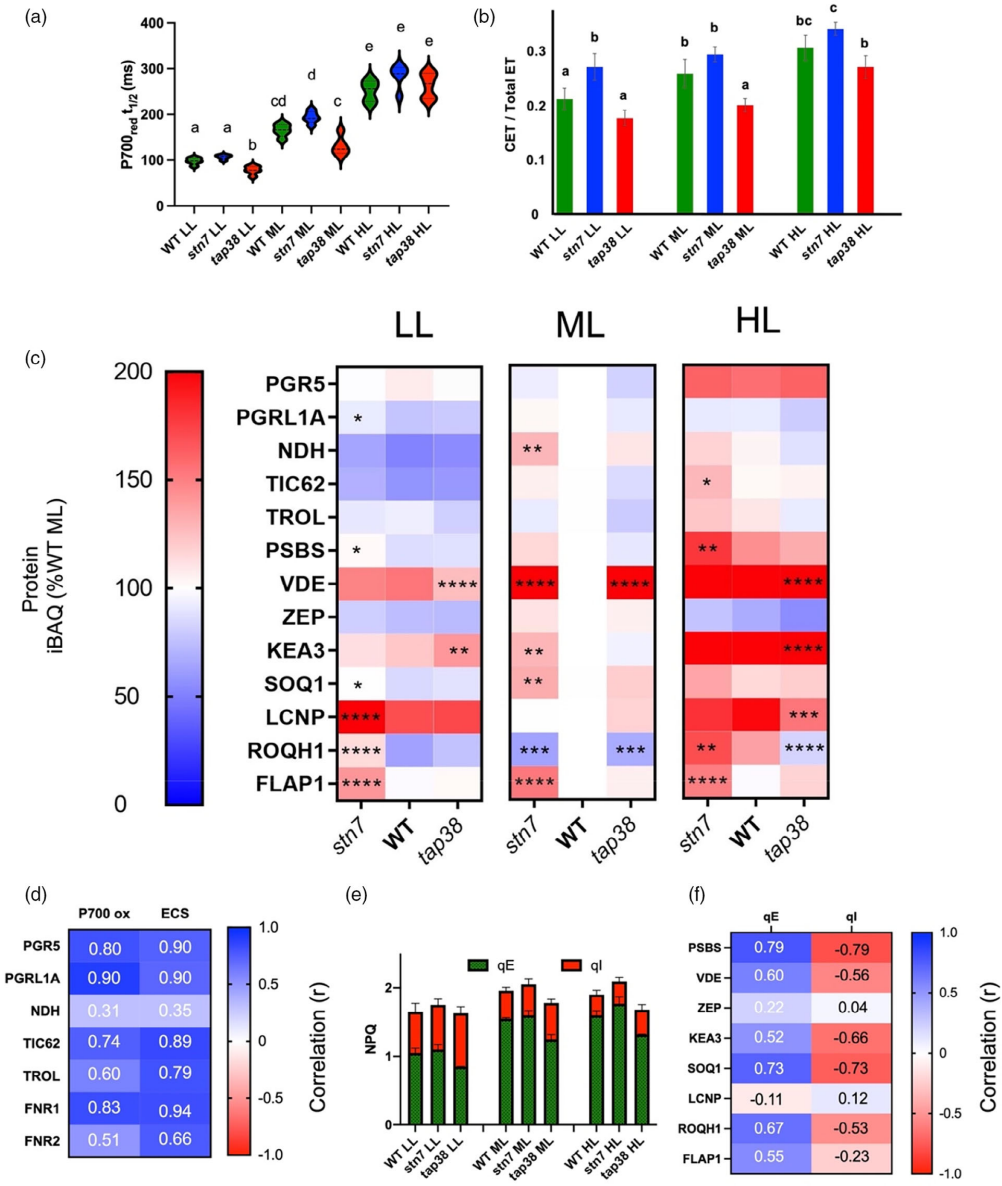
Assessment of changes in cyclic electron transfer (CET) and non‐photochemical quenching (NPQ) during developmental acclimation. (a) P700 oxidation half‐time upon illumination with 255 μmol photons m^−2^ s^−1^ 740 nm light (estimation of CET; Joliot & Joliot, 2002) with pairwise significant differences calculated using one‐way anova with Tukey's multiple comparisons test: a/b (*P* = 0.011), a/c (*P* ≤ 0.0001), a/d (*P* ≤ 0.0001), a/e (*P* ≤ 0.0001), b/c (*P* = 0.021), b/d (*P* ≤ 0.0001), b/e (*P* ≤ 0.0001), c/d (*P* = 0.0099), c/e (*P* ≤ 0.00010, and d/e (*P* = 0.015). (b) CET estimated as a proportion of total electron transfer using the ECS method (Kramer et al., 2021) with pairwise significant differences calculated using one‐way anova with Tukey's multiple comparisons test: a/b (*P* = 0.0013), a/c (*P* ≤ 0.0001), and b/c (*P* = 0.0099). (c) Heatmap of MS analysis showing the relative abundance of CET‐ and NPQ‐related proteins in WT, *stn7*, and *tap38* acclimated to LL, ML, and HL, expressed as a percentage of the WT at ML. The pixels are shaded and significant differences compared to WT are indicated by asterisks as described in Figure 2. (d) Pearson correlation of the CET activity measured by ECS and P700^+^ oxidation half‐time methods with protein iBAQ values of CET‐related proteins. (e) qE and qI measured following 5 min of illumination at 500 μmol photons m^−2^ s^−1^ 635 nm light. (f) Pearson correlation of the maximum qE and qI values with iBAQ values of NPQ‐related proteins. Blue panels indicate a positive correlation while red panels indicate a negative correlation.

Hepworth et al. (2021) and Strand et al. (2013) have previously demonstrated that CET levels are affected in *stn7* and *tap38*. Here we used similar spectroscopic methods to compare CET activity in WT and mutant plants grown at different irradiances. The rate of P700 oxidation under 740 nm illumination (255 μmol photons m^−2^ s^−1^) provides an indication of the potential for CET (Joliot & Joliot, 2002). As shown previously, P700 oxidation half‐time (P700 t½) increases in plants as a function of growth light intensity, consistent with a higher capacity for CET (Flannery et al., 2021a). Figure 4a shows that LL and ML grown WT and *stn7* plants had increased capacity for CET compared to *tap38*, but in HL grown plants this difference disappeared. These differences in P700 t½ could not be attributed to differences in LHCI (i.e. far‐red absorbing) antenna size between *stn7* and *tap38*, since these were similar (Figure 3a). Since PC levels (Figure 2b) are also similar in both mutants, differences in donor side limitation cannot easily explain the differences either. An alternative estimate of CET is derived from comparison of the rate of decay of the electrochromic shift (ECS) measured by the absorption difference at 515–550 nm on whole leaves. In principle, after a steady state is reached in the light, the initial rate of ECS decay upon light‐to‐dark transition will be proportional to the electron transfer rate in the preceding light period. The rate of decay following illumination with light preferentially exciting PSI (740 nm) gives the rate of CET, while light exciting both PSI and PSII (635 nm) stimulates both CET and LET (Kramer et al., 2021). Using this approach, we found the CET is larger as a proportion of total electron transfer in *stn7* in LL grown plants compared to WT and *tap38.* CET is also larger in *stn7* ML and HL grown plants compared to *tap38*, consistent with previous observations by Hepworth et al. (2021) (Figure 4b).

We were able to further rationalise this spectroscopy‐based evidence at the proteomic level with increases in several CET‐related proteins observed in *stn7*. Two pathways of CET exist in plants, one mediated by Proton Gradient Regulation 5 (PGR5), which is associated with PGRL1, the other by the NADH dehydrogenase‐like photosynthetic complex I (NDH) (Yamori & Shikanai, 2016). As shown in Figure 4c, in the WT PGR5 abundance is similar at LL and ML but increases in HL; however, no significant differences are observed between the WT and mutants. PGRL1 abundance decreases in LL in the WT but is steady between ML and HL. NDH abundance decreases in LL in the WT but is unchanged between ML and HL. TIC62 and TROL act as membrane tethers for FNR and have also been suggested to play a role in mediating the partitioning of electrons between LET and CET (Kramer et al., 2021). TIC62 abundance decreased in LL in the WT but was unchanged between ML and HL. TROL abundance was largely unchanged in the WT with growth light intensity. In contrast, *stn7* shows significantly higher levels of PGRL1 in LL, NDH in ML, and TIC62 in HL; however, *tap38* was not significantly different from the WT. Positive correlations above *r* = 0.85 were found between ECS and P700 t½ and the relative abundance of PGR5, PGRL1A, and FNR1 (Figure 4d).

In addition to the CET‐related proteins described above, Figure 4c shows the relative abundances of NPQ‐related proteins. In WT, the level of PSBS, a putative sensor of ΔpH that triggers the rapidly relaxing component of NPQ known as qE (Li et al., 2000), increased with growth irradiance as previously reported (Flannery et al., 2021a; Tikkanen et al., 2006). PSBS abundance was markedly increased in *stn7* in LL and HL, whereas *tap38* was similar to WT. In contrast, the abundance of violaxanthin de‐epoxidase (VDE), which converts the LHCII‐bound xanthophyll violaxanthin to zeaxanthin to enhance the sensitivity of qE to ΔpH (Murchie & Ruban, 2019), was increased in both LL and HL compared to ML in the WT, with increased abundance seen in both *stn7* and *tap38* under ML. The abundance of zeaxanthin epoxidase (ZEP), which performs the reverse reaction, decreased in both LL and HL relative to ML, though the amounts were similar between the WT and the mutants. KEA3, a putative H^+^/K^+^ antiporter that has been shown to affect the relaxation of qE (Armbruster et al., 2014), increased strongly in abundance in HL and showed a moderate increase in LL, although the abundance only significantly deviated in *stn7* in LL and ML. The SOQ1, LCNP, and ROQH1 proteins have been found to affect the capacity for a slowly relaxing component of NPQ known as qH (Amstutz et al., 2020; Brooks et al., 2013; Malnoë et al., 2018). In the WT, SOQ1 and ROQH1, which suppress qH, show increased relative abundance in HL and a decrease in LL, whereas the abundance of LCNP, which stimulates qH, increased in both LL and HL compared to ML. In LL, SOQ1, LCNP, and ROQH1 levels were higher in *stn7* compared to the WT, with SOQ1 and ROQH1 levels also significantly different at ML. On the other hand, in *tap38* only at HL were the differences significant, with less LCNP and ROQH1 compared to the WT. NPQ capacity was divided into qE and qI components in the WT and mutants by exposing plants to 500 PAR with the amount of NPQ relaxing after 5 min quantified as qE and that which remained classified as qI (Figure 4e). Significantly decreased capacity for qE was observed in *tap38* plants relative to WT under all growth irradiances (Figure 4e); in contrast, qI capacity decreased with growth irradiance but here levels were similar between the WT and mutants. A positive (*r* ≤ 0.79) correlation exists between PSBS and qE capacity and a similar negative correlation with qI capacity was observed. Finally, FLAP1 has also been implicated in the regulation of NPQ, since mutants lacking this protein have slightly higher levels of NPQ (Sato et al., 2017). Relative abundance of FLAP1 was similar across all growth irradiances in the WT and *tap38*, though elevated in *stn7* (Figure 4c). However, FLAP1 abundance was poorly correlated with both qE and qI (Figure 4f).

### Increased thylakoid grana diameter and stacking are observed in *stn7* plants grown under LL

**Figure 5:**
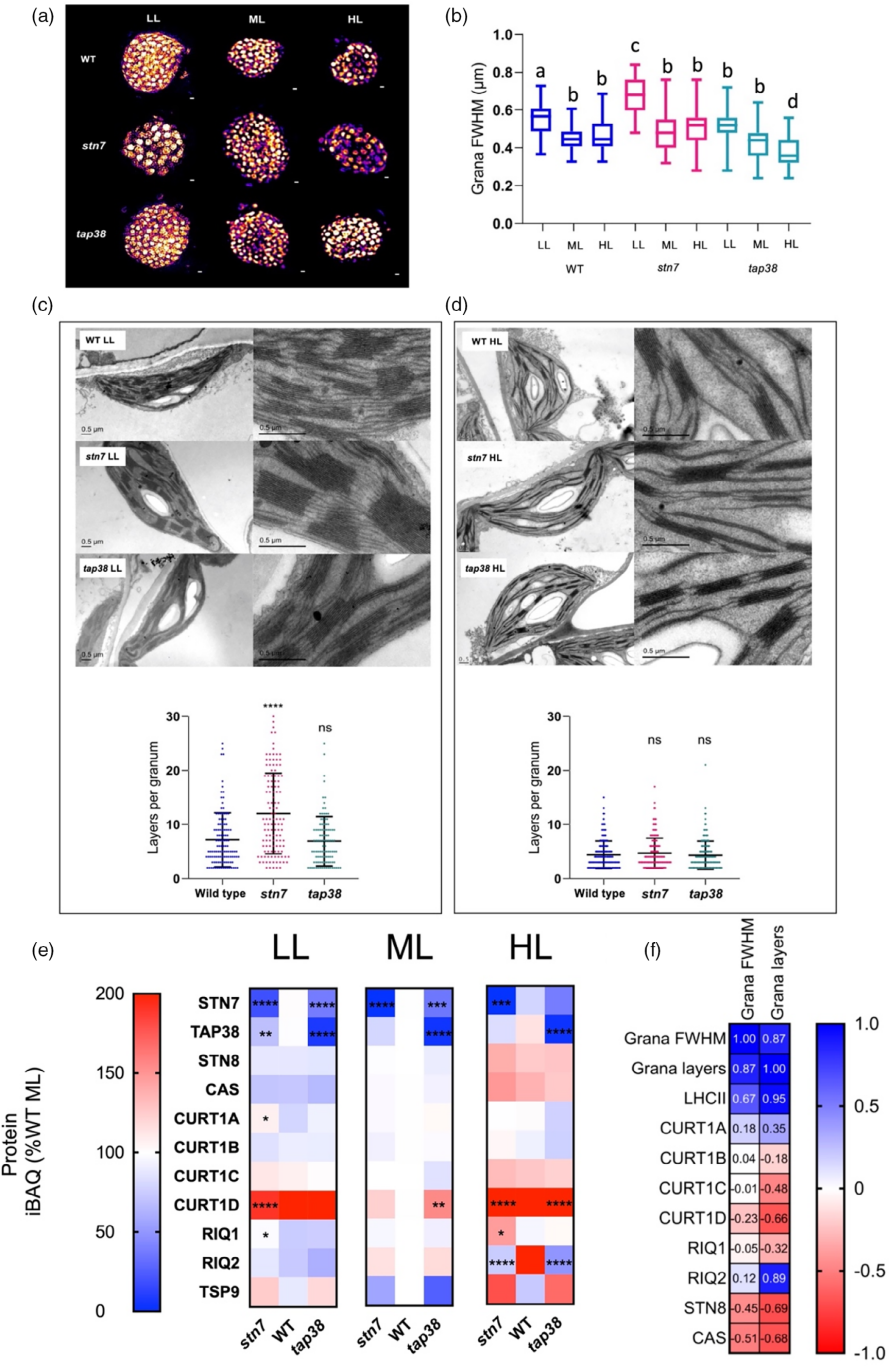
Assessment of changes in thylakoid membrane grana size during developmental acclimation. (a) 3D‐SIM images (shown as Max Projections on the *z*‐axis with tricubic sharp interpolation) of chloroplasts in WT, *stn7*, and *tap38* plants acclimated to LL, ML, and HL (scale bar: 0.5 μm). (b) Full width at half‐maximum (FWHM) fluorescence intensity of the fluorescent spots (grana) in 3D‐SIM images of chloroplasts in LL (*n* = 67), ML (*n* = 84), and HL (*n* = 71) leaves. Data are presented as mean ± SD. Pairwise significant differences were calculated using one‐way anova with Tukey's multiple comparisons test: a/b (*P* = 0.034), a/c (*P* = 0.017), a/d (*P* = 0.044), b/c (*P* = 0.009), b/d (*P* = 0.047), and c/d (*P* ≤ 0.0001). (c) Thin‐section electron micrographs of chloroplasts in WT, *stn7*, and *tap38* acclimated to LL (scale bar: 0.5 μm). Graph shows the number of membrane layers per grana stack calculated from electron microscopy images of chloroplasts in LL WT (*n* = 116), *stn7* (*n* = 121), and *tap38* (*n* = 102) (one‐way anova with Tukey's multiple comparisons). ****P < 0.0001. Data are presented as mean ± SD. (d) Thin‐section electron micrographs of chloroplasts in WT, *stn7*, and *tap38* acclimated to HL (scale bar: 0.5 μm). Graph shows the number of membrane layers per grana stack calculated from electron microscopy images of chloroplasts in HL Col‐0 (*n* = 332), *stn7* (*n* = 192), and *tap38* (*n* = 185) (one‐way anova with Tukey's multiple comparisons). ****P < 0.0001. Data are presented as mean ± SD. (e) Heatmap of MS analysis showing the relative abundance of grana stacking‐related proteins in WT, *stn7*, and *tap38* acclimated to LL, ML, and HL, expressed as a percentage of the WT mean at ML. The pixels are shaded and significant differences compared to WT are indicated by asterisks as described in Figure 2. (f) Pearson correlation of the mean number of membrane layers per granum and grana FWHM with iBAQ values of grana stacking‐related proteins. Blue panels indicate a positive correlation while red panels indicate a negative correlation.

The LTR to irradiance is known to affect the thylakoid macrostructure, with HL growth favouring narrower diameter grana with fewer membrane layers, while LL favours the opposite i.e. increased stacking and diameter (Anderson, 1986; Flannery et al., 2021a; Walters, 2005). Using structured illumination microscopy (SIM), we compared the grana diameter of chloroplasts in WT, *stn7*, and *tap38* leaves (Figure 5a,b). Previous results have shown absence of STN7 increases grana diameter, while loss of TAP38 has the opposite effect (Armbruster et al., 2013; Fristedt et al., 2009b; Wood et al., 2019). We found *stn7* chloroplasts had wider grana in LL grown plants, whereas *tap38* grana were narrower in LL and HL grown plants compared to both WT and *stn7*. Determination of grana structure by thin‐section electron microscopy (EM) revealed that *stn7* had an increased number of layers per grana in LL (Figure 5c), although it was similar to the WT under HL (Figure 5d). However, *tap38* was not significantly different from WT in either case. The relative abundance of STN7 and TAP38 calculated by LFQ‐MS analysis compared to the WT are shown in Figure 5e. The *stn7* mutant contained undetectable amounts of STN7 under ML and only around 2% of its WT level at HL. At LL, STN7 was present at around 20% of that seen in WT; this may explain the appearance of a faint band corresponding to the PSI–LHCI–LHCII supercomplex seen by BN‐PAGE (Figure 3b). It is possible that the location of the T‐DNA insertion in an intron of *stn7* may allow some residual expression in the mutant. Notably, given the proposed role of STN7 in signalling and regulation, this protein was also significantly depleted in *tap38*, being present at around 35% of WT levels in all light conditions, consistent with Wunder et al. (2013) and Trotta et al. (2016). The corresponding effect was observed, albeit to a much lesser extent, in *stn7*; i.e. the loss of STN7 caused a significant reduction in the amount of TAP38 in LL plants. In *tap38*, where the T‐DNA insertion is located within the 5′ region of the gene, the phosphatase was depleted to less than 10% of WT levels in all light conditions. On the other hand, the PSII kinase STN8 and the calcium sensor kinase CAS displayed similar patterns of light acclimation in both WT and mutants.

In addition to LHCII abundance and phosphorylation state, grana structure has been shown to be affected by the levels of the thylakoid curvature (CURT1A–D) and Reduced Induction of NPQ (RIQ1 and RIQ2) proteins (Armbruster et al., 2013; Pribil et al., 2018; Yokoyama et al., 2016). As shown in Figure 5e, LFQ‐MS quantification indicated that CURT1A and CURT1B levels were not appreciably affected by light intensity in WT, while CURT1C and CURT1D levels appeared to increase under HL, while the latter also increased under LL. This pattern was also reflected in the mutants, although there was significantly more CURT1A in *stn7* in LL and more CURT1D in *stn7* in LL and *tap38* in ML. In WT, both RIQ1 and RIQ2 levels decreased under LL to a similar extent, and while RIQ1 maintained its ML level under HL, RIQ2 levels increased. In LL and HL, RIQ1 abundance was significantly higher in *stn7* than in WT. In contrast, RIQ2 abundance was lower in both mutants in HL compared to the WT. Figure 5f shows the expected positive correlations between grana diameter, the number of grana layers per stack, and LHCII abundance. Of the CURT1 and RIQ proteins described above, only RIQ2 appears to correlate with the number of grana layers.

TSP9 is another identified phosphorylation target of STN7 and has been proposed as a link between STN7 and the LTR (Fristedt et al., 2008). In particular, it may play a role in acclimation to HL, since exposure of mutants lacking TSP9 to HL does not induce expression of a group of signalling proteins that are normally upregulated after HL treatment (Fristedt et al., 2009a). The MS analysis showed that TSP9 abundance is influenced by growth light intensity and genotype. In WT plants, TSP9 decreased in HL but was unchanged between LL and ML (Figure 5e). Interestingly, when grown at HL, both *stn7* and *tap38* thylakoids contained significantly more TSP9. However, since phosphorylation of TSP9 induces its dissociation from the stromal surface of the thylakoid membrane (Carlberg et al., 2003), these values may represent binding of the protein rather than absolute abundance. If STN7 is responsible for this phosphorylation that leads to release of TSP9 from the membrane, a lack of STN7 in the *stn7* mutant, and to a lesser extent *tap38*, might cause more TSP9 to be bound to the membrane in HL (Figure 5e).

## DISCUSSION

Building on our earlier characterisation of the proteomic changes associated with developmental acclimation to changes in white light intensity and indoor versus outdoor growth environments (Flannery et al., 2021a, b), here we addressed the role of STN7 and its cognate phosphatase TAP38 in the LTR. Previous work showed that *stn7* plants, unlike the WT, were unable to change their PSI/PSII ratio, LHCII/PSII ratio, or chlorophyll *a*/*b* ratio in response to varying spectral quality (Bonardi et al., 2005; Pesaresi et al., 2009). This result led the authors to suggest that STN7 plays a central role in the LTR signalling pathway, phosphorylating an unidentified protein to trigger the changes in nuclear and chloroplast gene expression required to tailor the thylakoid proteome to the prevailing light environment. In contrast, our results clearly demonstrate that the LTR is retained in the *stn7* mutant, e.g. CO_2_ assimilation capacity increases with growth light intensity, grana diameter and stacking decrease, and PSI and LHCII levels decrease in HL and increase in LL, whereas RuBisCO, cyt*b*_6_ *f*, and ATP synthase levels decrease in LL and increase in HL, all patterns typical of the LTR (Anderson, 1986; Anderson et al., 1988; Bailey et al., 2001; Bailey et al., 2004; Boardman, 1977; Schöttler & Tóth, 2014; Walters, 2005). Therefore, it is unlikely that STN7 is an essential component of the signalling cascade which accompanies the LTR. Similarly, the *tap38* mutant shows the ability to tune these parameters in response to growth light intensity and therefore we conclude TAP38 also holds no crucial role in the LTR. Our data could be reconciled with past work (Pesaresi et al., 2009) if one assumes that STN7‐based signalling is only essential to adapt to changes in light quality e.g. the acclimation to natural shade. Alternatively, STN7 may exert its effect only indirectly through modulation of the PQ redox state by controlling state transitions and dynamic thylakoid stacking.

While the LTR remained present in *stn7* and *tap38* plants, there were nevertheless a series of thylakoid proteomic changes compared to the WT and one another, though these appear to be better correlated with the altered electron transfer chain redox state observed in the mutants. In the WT, the LL and ML growth conditions we employed favoured State II, since a significant proportion of PSI in our BN‐PAGE was present as the PSI–LHCI–LHCII supercomplex, whereas the HL growth conditions were more tilted towards State I. Since *stn7* is locked in State I (Bellafiore et al., 2005), the PSI–LHCI–LHCII supercomplex is largely absent in all growth light conditions. Consistent with this, PSII remains relatively overexcited and therefore the value of 1 − qP (a proxy for the extent of PQ pool reduction) remains higher in LL and ML grown stn7 plants at 25 PAR compared to the WT, although it is similar at 500 PAR. The tweaked LTR of the *stn7* mutant to this additional redox pressure on PSII is to increase the abundance of cyt*b*_6_ *f*, PSI, and LHCII trimers, particularly the LHCB2 component, which in the WT is required for the formation of the PSI–LHCI–LHCII supercomplex (Pan et al., 2018). In addition, *stn7* increases the abundance of the rapidly relaxing NPQ components PSBS and VDE, as observed previously (Grieco et al., 2012; Tikkanen et al., 2006), in addition to proteins involved in slowly relaxing NPQ including SOQ1, LCNP, and ROQH1, likely in an attempt to further mitigate the excitation pressure on PSII. While a higher PSII limitation in *stn7* was expected, we also noted that there was an increase in PSI acceptor side limitation in LL, ML, and HL in *stn7* plants exposed to 25 PAR (Figure 1e). This result is consistent with our previous observations in stn7 and suggests the increased CET activity we observe in this mutant compared to the WT may be a response to this pressure (Hepworth et al., 2021). Enhanced CET in stn7 may be in part based on increases in the relative amounts of PGRL1A, NDH complex, TIC62, FNR1, and FNR2. Yet these adjustments in photosynthetic complex stoichiometry via the LTR in *stn7* plants fail to fully ameliorate the symptoms of redox pressure on PSII and PSI, since growth and CO_2_ assimilation remain lower compared to WT and *tap38* in LL and ML plants. Under HL growth, these issues may be largely resolved in *stn7* by the increased amount of light available for PSI. In contrast, *tap38* is locked in State II (Pribil et al., 2010; Shapiguzov et al., 2010) and thus the PSI–LHCI–LHCII supercomplex is present to similar extents in all growth light conditions. Therefore, these redox signals are dampened and consequently LL and ML grown *tap38* behaves more like WT, though PSI abundance increases less under LL. For LL and ML grown *tap38* plants, CO_2_ assimilation was significantly lower and PSI and PSII redox pressure was higher when measured at 500 PAR compared to both WT and *stn7* (Figure 1c,e; Figure S2). However, in HL grown plants these differences disappear, suggesting the LTR in *tap38* can compensate for the lack of TAP38‐dependent dephosphorylation under prolonged high growth irradiance. Consistent with this view, it was previously shown that some phosphatase activity towards LHCII (and possibly other phosphorylated STN7 targets) remains in the *tap38* mutant (Pribil et al., 2010; Shapiguzov et al., 2010). These observations suggest that for optimal growth under LL and ML, state transitions and dynamic thylakoid stacking are indispensable and the LTR has limited ability to compensate via adjustments in photosynthetic complex stoichiometry. In contrast, when light is abundant the LTR can better compensate. This idea is consistent with the convergence of the proteomes of the WT and two mutants under HL conditions (Figure 2a). In addition to the differences described above in the proteomic responses of *stn7* and *tap38* compared to the WT, there were also a significant number of similarities. The levels of a diverse range of chloroplast proteins involved in functions including electron transfer, light harvesting regulation, the Calvin cycle, and grana stacking (e.g. RuBisCO, RBCA, PC, VDE, ROQH1, CURT1D, and TSP9) behaved in an almost identical way in the two mutants. One possibility is that this might represent a general stress response, possibly involving reactive oxygen species (ROS) brought about by the slightly unbalanced electron transfer in the mutants and associated effects on redox homeostasis as suggested previously (Tikkanen et al., 2012).

In conclusion, we found no evidence in this study that STN7 or TAP38 is essential for the LTR in Arabidopsis to white light intensity. The deviations from the typical WT acclimation pattern are correlated with the disparate redox states of *stn7* and *tap38* mutants. Pesaresi et al. (2009) discounted state transitions as having a major influence on the LTR since Arabidopsis plants lacking PSAL, which cannot bind LHCII to PSI in State II, still showed acclimation to light quality. However, dynamic thylakoid stacking remains in PSAL‐deficient plants and therefore they could still modulate the redox state of PSI upon shifts from LL to HL (Hepworth et al., 2021). Therefore, it is possible that PSI redox state is an overlooked factor in the signalling pathway for the LTR. Indeed, there is evidence that the redox state of the Trx/Fd system, which is reduced via electrons from PSI, is a key factor in shaping the LTR (Ibrahim et al., 2022; Lempiäinen et al., 2022), an idea which requires further investigation. The differences between the mutants and WT were largely abolished by growth in HL, which is consistent with the primary role of the STN7/TAP38 system in LL. The separate responses we observe to LL and HL are also consistent with previous results (Walters, 2005) and the varying effects of PQ oxidation and PQ reduction on leaf development (Zoulias et al., 2021). Retrograde signals from the chloroplast involving PQ oxidation affect transcript levels of the SPEECHLESS and MUTE transcription factors, which negatively regulate the stomatal index via the mitogen‐activated protein kinase (MAPK) pathway, while PQ reduction acts through the STOM factor to increase the stomatal index. These effects are independent of phyB, which also influences PSI/PSII ratios upon adjustment of the PQ redox state with varying spectral quality of growth light (Ibrahim et al., 2022). The LTRs to LL and HL therefore appear to involve both overlapping and distinct signalling pathways that are collectively integrated and do not rely upon a single sensing hub. For instance, both light spectral quality and intensity have been shown to independently affect leaf and chloroplast development (Walters & Horton, 1995a; Walters & Horton, 1995b). It is therefore likely that both leaf development and the stoichiometry of photosynthetic proteins within the thylakoid are regulated by incorporating a complex network of inputs from several signalling pathways, including PQ redox state, the Trx/Fd system, and phyB. The LTR system thereby provides the functional flexibility to achieve diverse and tailored developmental outcomes according to prevailing environmental conditions.

## METHODS

### Growth and acclimation of Arabidopsis

*Arabidopsis thaliana* WT Col‐0, *stn7* (SALK 073254), and *tap38* (SALK 025713), the latter two mutants previously successfully complemented with the WT proteins (Bellafiore et al., 2005; Pribil et al., 2010), were grown on John Innes M3 compost mixed at a 4:1:1 ratio with perlite and vermiculite in a Conviron plant growth room under fluorescent bulbs (emission spectrum shown in Figure S1) at 60% relative humidity, at 21°C in the daytime and 15°C at nighttime, at a light intensity of 150 μmol photons m^−2^ s^−1^ with a 12‐h photoperiod for 2 weeks until rosettes reached a diameter of around 3 cm. The plants were then transferred to LL (25), ML (150), or HL (500 μmol photons m^−2^ s^−1^) by moving closer to or further from the light source; all other conditions remained the same, cabinet temperature regulation ensured that leaf temperature varied no more than ±2°C under the three light intensities, and plants were randomly arranged. Plants were acclimated for different lengths of time to account for faster maturation under higher light intensity. HL plants were harvested at 4 weeks, ML plants were harvested at 5 weeks, and LL plants were harvested at 7 weeks, with all plants harvested prior to flowering.

### Chlorophyll fluorescence and P700 absorption spectroscopy

Pulse amplitude modulated chlorophyll fluorescence was measured and P700 absorption spectroscopy was conducted using a Dual‐KLAS‐NIR photosynthesis analyser (Walz, Germany) (Klughammer & Schreiber, 2016) on LL, ML, or HL plants dark‐adapted for 1 h. Maximum P700 absorption was determined using a 300‐ms saturating pulse of 18 000 μmol photons m^−2^ s^−1^ in the presence of 255 μmol photons m^−2^ s^−1^ far‐red light (740 nm). The intensity of far‐red light used is given according to the values provided on Walz Dual‐KLAS NIR software. Chlorophyll fluorescence parameters and relative P700 redox state were determined at each light intensity using 12 μmol photons m^−2^ s^−1^ modulated measuring light (540 nm) in combination with a saturating pulse of 18 000 μmol photons m^−2^ s^−1^. Actinic light was provided by 635 nm LEDs. Fluorescence parameters were calculated according to Maxwell and Johnson (2000) and P700 parameters were calculated according to Klughammer and Schreiber (1994). Relative CET capacity was assessed using the method of Joliot and Joliot (2002) (Figure 4a). Briefly, dark‐adapted plants were given a 200‐ms flash of 635 nm light (2000 μmol photons m^−2^ s^−1^), followed by 5 sec of dark, before being illuminated with 255 μmol photons m^−2^ s^−1^ far‐red light (740 nm) to induce PSI oxidation. The half‐time for the rise in P700 oxidation from the moment far‐red light illumination started was taken as a measure of CET efficiency. Longer half‐times, i.e. delayed P700 oxidation, reflected more efficient CET. An alternative measure of CET was provided by the method of Kramer et al. (2021). Briefly, plants were dark‐adapted for 1 h and then illuminated for 20 sec in the presence of 255 μmol photons m^−2^ s^−1^ far‐red light (740 nm), and the rate of decay of the ECS signal (ΔA515–550) was used a measure of CET and compared to the ECS generated by a subsequent 1‐ms saturating pulse of 18 000 μmol photons m^−2^ s^−1^ (635 nm light) to provide a measure of total electron transfer (LET + CET).

### Infra‐red gas exchange analysis

CO_2_ assimilation was measured using an LI‐6800 portable photosynthesis system (LiCor, Lincoln, NE, USA) on mature leaves attached to the plants during the middle of the day, that is, from 3 to 6 h into the photoperiod. Relative humidity of the chamber (6 cm × 6 cm) was maintained at 60%, the flow rate was 150 μmol s^−1^, and the block temperature was 20°C. Sample CO_2_ was maintained at 400 ppm. Leaves were acclimated to 25 or 500 μmol photons m^−2^ s^−1^ of light at 635 nm for 20 min prior to each measurement to ensure stomata were open and at their normal aperture size prior to measurement and photosynthesis was at steady state.

### Electron microscopy of leaf thin sections

Leaf discs were taken at the point of harvest from positions in the centre of exposed leaves. Electron micrographs of leaf thin sections were obtained according to Wood et al. (2018).

### Structured illumination microscopy

Samples were prepared, imaged, and analysed according to Wood et al., 2019.

### Isolation of thylakoid membranes

Thylakoid membranes were isolated according to Albertsson et al. (1994), with the addition of 10 mm NaF to all buffers.

### Chlorophyll analysis

Absorption spectra were taken on an Agilent Technologies Cary 60 UV‐VIS spectrophotometer. Chlorophyll concentrations and chlorophyll *a*/*b* ratios were determined according to Porra et al. (1989).

### SDS‐PAGE and ProQ‐Diamond phosphoprotein staining

Thylakoids (5 mg of chlorophyll) were solubilised in Laemmli buffer and separated by SDS‐PAGE on a 12% (w/v) Bis‐Tris gel in MOPS buffer (Benson et al., 2015). Diamond Pro‐Q Phospho staining (Life Technologies) was performed as previously described by Mekala et al. (2015).

### BN‐PAGE

Stromal lamellae were solubilised at 0.5 mg mL^−1^ chlorophyll in 2% digitonin, 50 mm Bis‐Tris pH 7.2, 10 mm NaF, and 10% glycerol for 1 h on ice. Grana membranes were solubilised in 1.0% *n*‐dodecyl α‐d‐maltoside, 50 mm Bis‐Tris pH 7.2, 10 mm NaF, and 10% glycerol for 1 h on ice. Solubilised protein complexes were resolved by BN‐PAGE, as previously described (Wood et al., 2019).

### Quantitative proteomic analysis of thylakoid membranes

Thylakoid membrane proteins were solubilised and digested with a combination of endoproteinase Lys‐C and trypsin in 1% (w/v) sodium laurate and 100 mm triethylammonium bicarbonate pH 8.5 as described previously (Lin et al., 2013). Additional sample processing and analysis by nanoLC‐MS were performed according to Flannery et al. (2021a). MaxQuant v. 1.6.3.4 was used for mass spectral data processing and protein identification with the iBAQ (Schwanhäusser et al., 2011) label‐free quantification option selected and other parameters as previously specified (Flannery et al., 2021a). iBAQ abundance scores were processed using Perseus v. 1.6.2.3 (Tyanova et al., 2016) by normalisation to the intra‐analysis sum of the iBAQ abundance scores of 39 subunits mapping to PSII, PSI, cyt*b*_6_ *f*, and ATP synthase, as previously implemented by Flannery et al. (2021a).

## AUTHOR CONTRIBUTIONS

SEF, MJD, SAC, CNH, PJJ, and MPJ designed the experiments. SEF, PJJ, FP, and TZE‐M performed the experiments. PJJ, MJD, SEF, SAC, CNH, and MPJ wrote the manuscript. All authors proof‐read and approved the manuscript.

## CONFLICT OF INTEREST

The authors have no conflict of interest to declare.

## Supporting information


**Figure S1.** Emission spectrum of fluorescent growth lights.
**Figure S2.** Additional chlorophyll fluorescence and P700 parameters. (a) The fraction of closed PSII reaction centres (1 − qP). (b) Non‐photochemical quenching (NPQ). (C) PSI quantum yield (Y(I)). (D) PSI donor side limitation (Y(ND)). All values were measured at either 25 or 500 μmol photons m^−2^ s^−1^ (PAR) using 635 nm LEDs. * denotes significant differences with respect to WT at each light intensity as determined by a modified Welch's *t*‐test (*q* < 0.05).
**Figure S3.** Total phosphoprotein staining. (a) Solubilised thylakoids were separated by SDS‐PAGE and subjected to ProQ Diamond Phosphoprotein staining. (b) Total protein staining by Coomassie to show equal loading in panel (a).

**Table S1.** Intensity‐based absolute quantification (iBAQ) of Arabidopsis thylakoid proteins. Comparison of *stn7* and *tap38* mutants and WT plants grown under low, moderate, and high light.

## Data Availability

The mass spectrometry proteomics data have been deposited to the ProteomeXchange Consortium via the PRIDE partner repository (http://proteomecentral.proteomexchange.org) with the data set identifier PXD037358. All other data can be obtained from the corresponding authors upon request.
