# Supplementary material for: STN7 is not essential for developmental acclimation of Arabidopsis to light intensity
Source: Plant J. 2023 Apr 11;114(6):1458–74. doi: 10.1111/tpj.16204 (PMC10952155; doi:10.1111/tpj.16204)
Supplement: Supplementary file 1 — Figure S1. Emission spectrum of fluorescent growth lights. Figure S2. Additional chlorophyll fluorescence and P700 parameters. (a) The fraction of closed PSII reaction centres (1 − qP). (b) Non‐photochemical quenching (NPQ). (C) PSI quantum yield (Y(I)). (D) PSI donor side limitation (Y(ND)). All values were measured at either 25 or 500 μmol photons m−2 s−1 (PAR) using 635 nm LEDs. * denotes significant differences with respect to WT at each light intensity as determined by a modified Welch's t‐test (q < 0.05). Figure S3. Total phosphoprotein staining. (a) Solubilised thylakoids were separated by SDS‐PAGE and subjected to ProQ Diamond Phosphoprotein staining. (b) Total protein staining by Coomassie to show equal loading in panel (a). [file TPJ-114-1458-s002.docx]

**
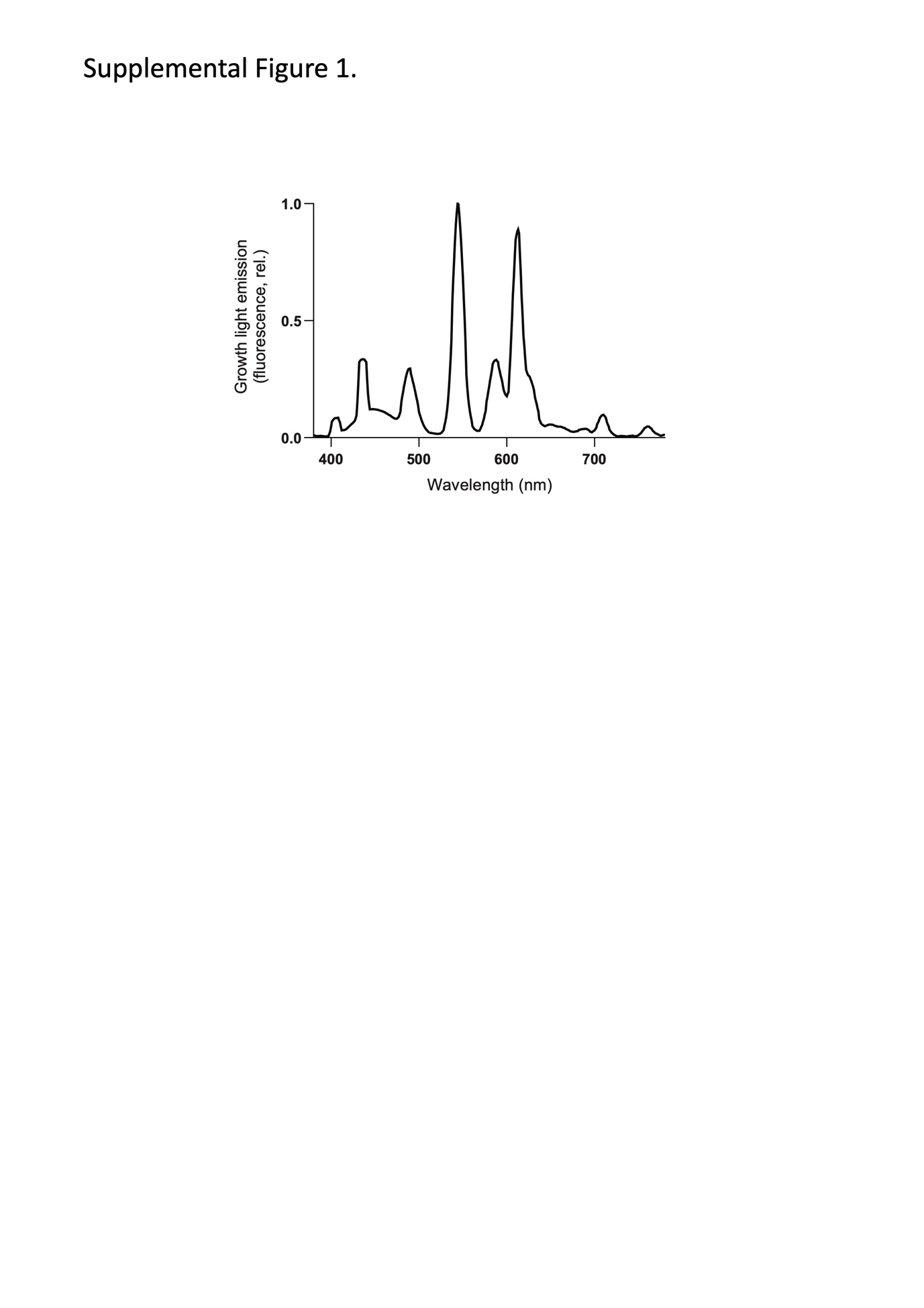
**

Emission spectrum of fluorescent growth lights.


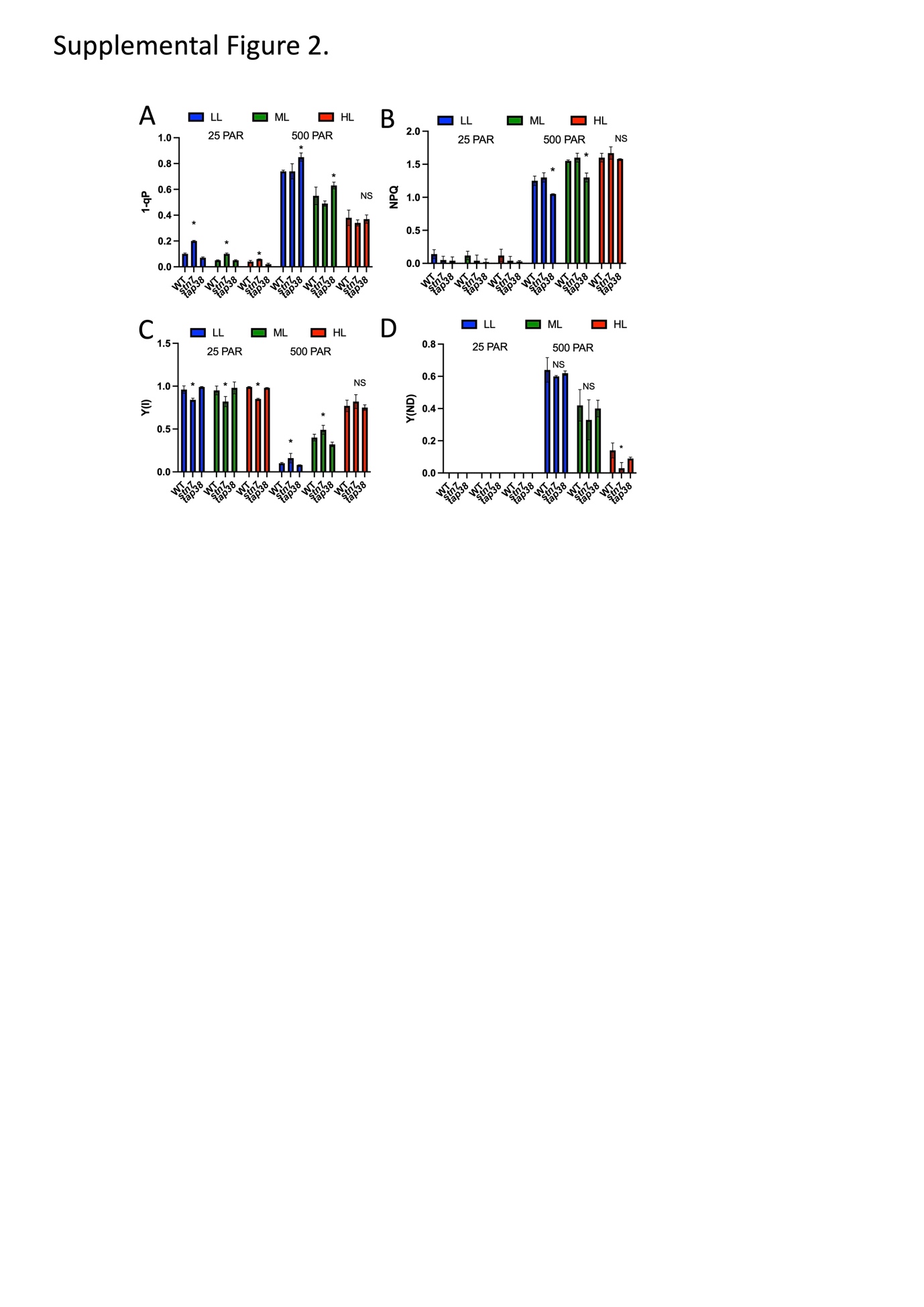


**Supplemental Figure 2. Additional chlorophyll fluorescence and P700 parameters. A,** The fraction of closed PSII reaction centres (1-qP). **B,** non-photochemical quenching (NPQ), **C**, PSI quantum yield (Y(I)), **D,** PSI donor side limitation (Y (ND)). All measured at either 25 or 500 µmol photons m^-2^ s^-1^ (PAR) using 635 nm LEDs. * denotes significant differences with respect to WT at each light intensity as determined by a modified Welch’s t-test (*q* < 0.05).


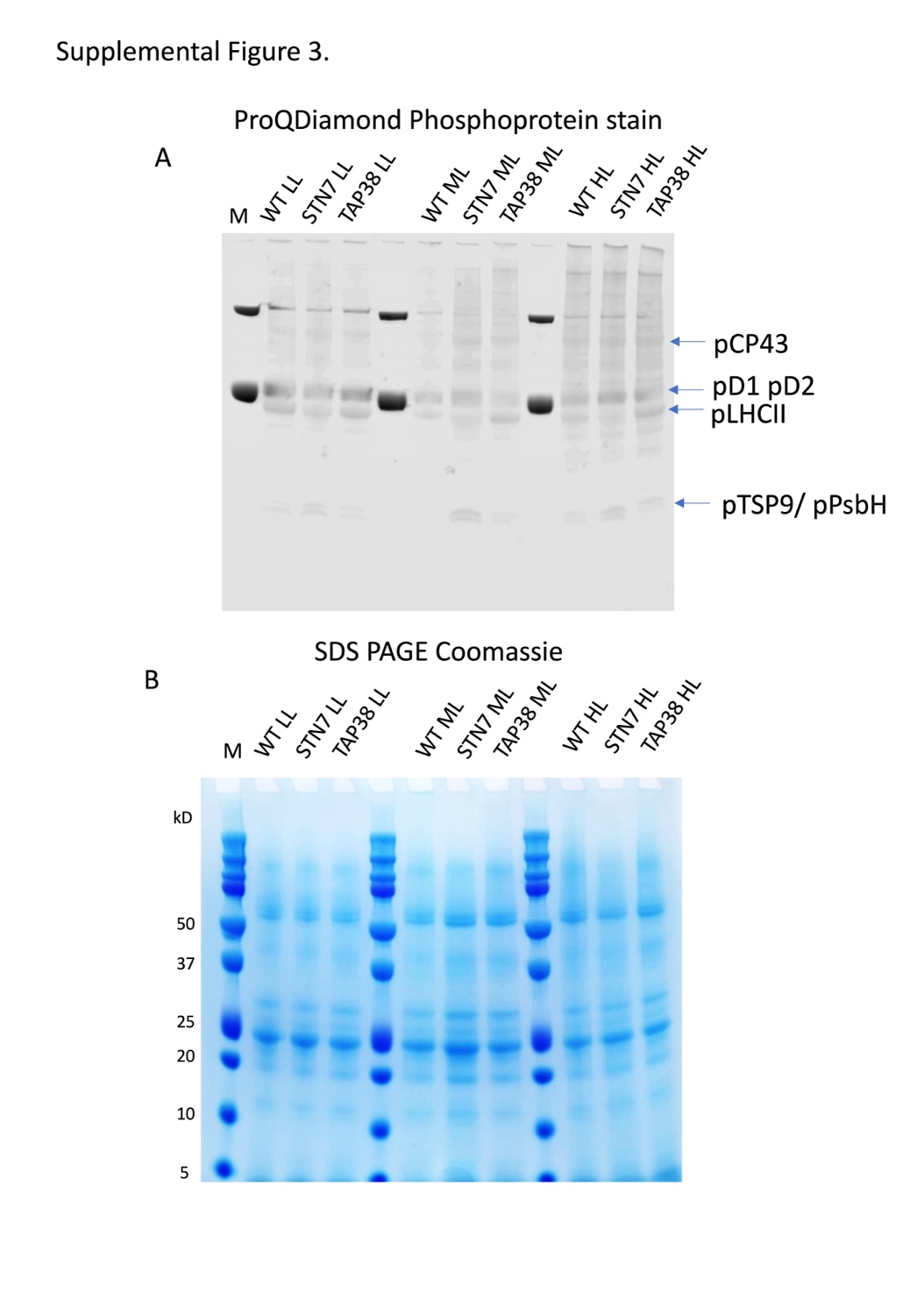


**Supplemental Figure 3. Total Phosphoprotein Staining. A**, Solubilised thylakoids were separated by SDS-PAGE and subjected to ProQ Diamond Phosphoprotein staining. **B**, Total protein staining by Coomassie to show equal loading in A.
